# A rapid and inexpensive method for the isolation and identification of *Giardia*

**DOI:** 10.1016/j.mex.2020.100998

**Published:** 2020-07-23

**Authors:** Zhangxia Lyu, Jianfan Wen

**Affiliations:** State Key Laboratory of Genetic Resources and Evolution, Kunming Institute of Zoology, Chinese Academy of Sciences, Kunming, Yunnan 650223, China

**Keywords:** *Giardia* isolation, *Giardia* identification, Trophozoite, Cyst

## Abstract

This work details a protocol for the isolation of *Giardia* species from their host animals and their identification based on their morphological and molecular characteristics. *Giardia* are intestinal protozoan parasites found in almost all vertebrates and the epidemiology of *Giardia* has attracted the attention of scientists due to their harm to humans and live stocks worldwide. *Giardia* trophozoites adhere to the surface of host's intestines using their ventral disc, and they also adhere the culture tube wall during in vitro culturing. We developed a method of isolating *Giardia* trophozoites according to this phenomenon, and a method of isolating *Giardia* cysts according to their special density as well. We validated the protocol by isolating and identifying *Giardia* species from their host animals, and all the results support that this methodology has a certain validity. It could help the further epidemiological researches and other researches requiring relatively pure living organism as materials of this harmful parasite.

• An isolation method with high efficiency which remains the physiological activity of isolated *Giardia*.
• An identification method with high accuracy which avoids the influences from other organisms.
• Low-cost, fast and convenient methodology.

## Graphical abstract

**Figure:**
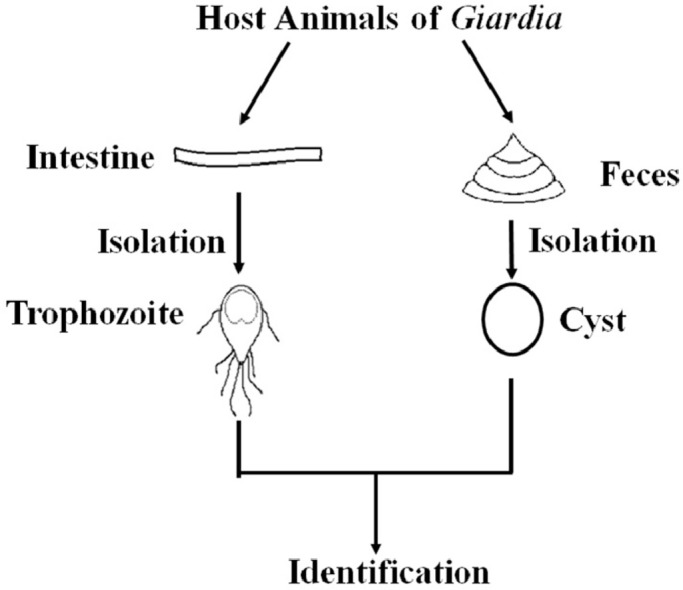

Specifications Table

**Table:** 

Subject Area:	Agricultural and Biological Sciences
More specific subject area:	Protozoan Parasite *Giardia*
Method name:	Isolation and identification of *Giardia* species
Name and reference of original method:	N/A
Resource availability:	GenBank, https://www.ncbi.nlm.nih.gov/genbank/. The JmodleTest software, http://jmodeltest.org. The clustalW 2.0 program, http://www.ebi.ac.uk/Tools/msa/. Phylogenetic analysis by maximum likelihood method, www.paml.com/. Commercial Kit (TaKaRa)

## Method details

### Background

Currently, the identification of *Giardia* was focused on *G. intestinalis* (Syn. *G. duodenalis* or *G. lamblia*) as it parasitizing humans and live stocks, the researches on other *Giardia* species were neglected at least partly due to the absence of isolation and identification method. The identification of *G. intestinalis* is usually based on molecular methods using the following five genes: *glutamate dehydrogenase, beta-giardin, elongation factor-1 alpha, triose phosphate isomerase* and *small subunit rRNA* (*SSU rRNA*) [1]. The current molecular methods of the identification of *Giardia* required scientist to extract DNA from homogenized faeces using commercial kit [2], which may be expensive for developing regions. Furthermore, there are no methods to directly isolate living *Giardia* trophozoites from their host animals at present. Thus, we developed a new method by which all *Giardia* species can be isolated from their host animals and then identified from their host animals. We have isolated and identified a new *Giardia* species successfully using this method, *G. cricetidarum* [3]. And we have also used this method to investigate the prevalence of *G. agilis* [4]. This is a cheap and fast method, which can help us to do the epidemiological researches and researches requiring relatively pure living organism as materials. in all *Giardia* species.

### Isolation of Giardia trophozoites and cysts

All collections of animal samples should follow local laws and ethics, and the experimental procedures and animal care conditions should be approved by the Ethic Committee of institutes. The animal samples should be anesthetized using appropriate anesthetics which induce rapid unconsciousness and death without pain or distress.

**Fig. 1:**
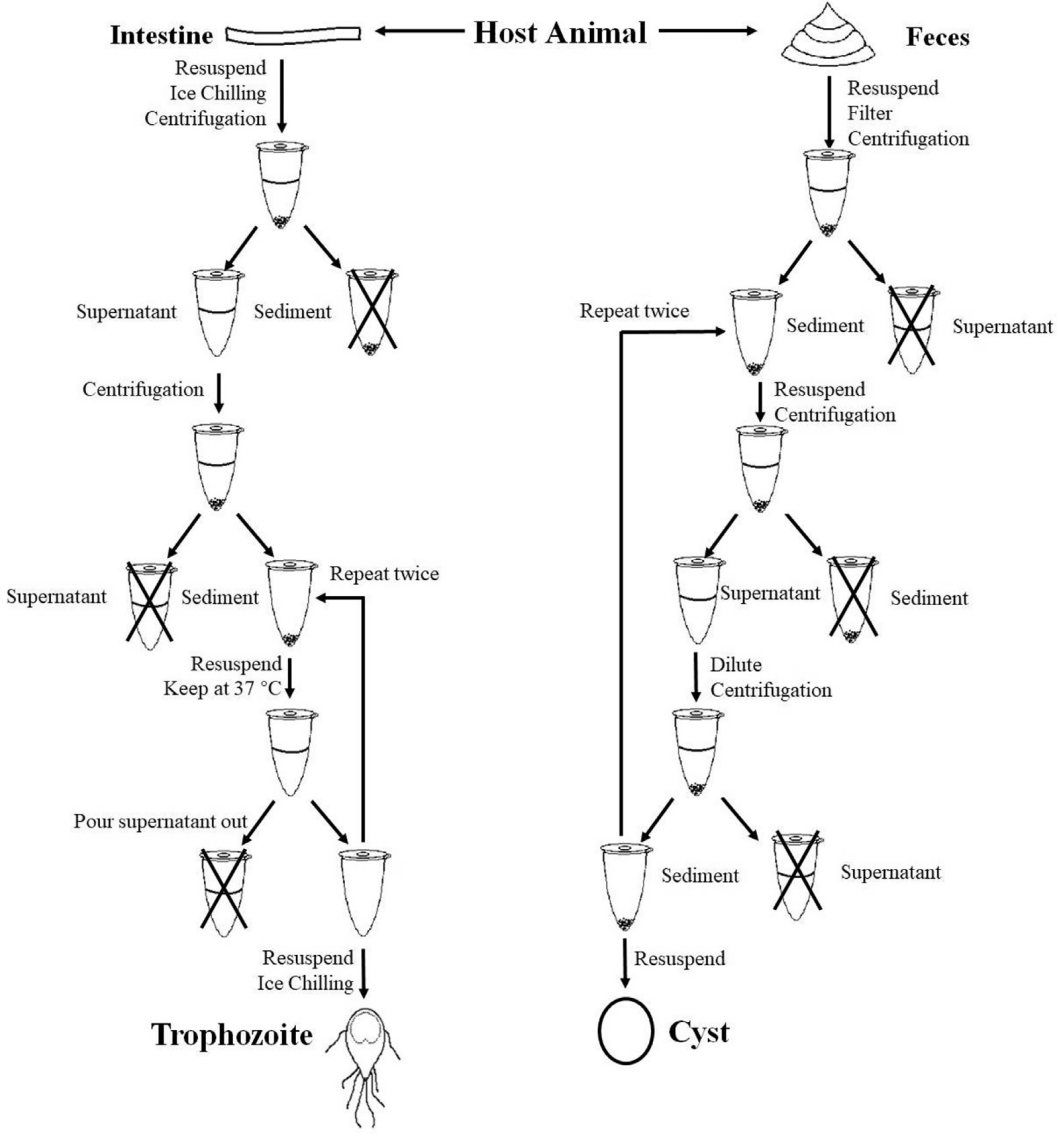
Schematic of the isolation of *Giardia* trophozoites and cysts from their host organisms.

**Fig. 2:**
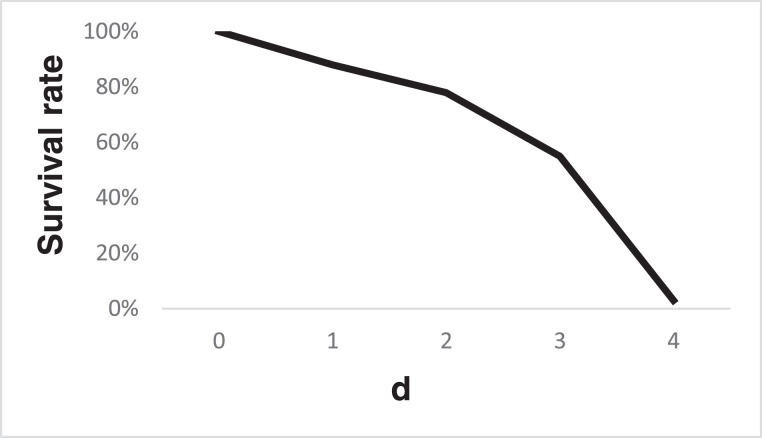
Survival rate of trophozoite samples stored at 4 °C.

*Giardia* trophozoites are collected from intestines of host animals. The animal samples are anesthetized using appropriate anesthetics and put to death without pain or distress. Then their intestines are taken out and cut into 0.1 cm segments. The segments of intestines are transferred into a centrifuge tube with adapted TYI-S-33 medium (please use 0.65% sodium chloride solution instead all of the adapted TYI-S-33 medium when the animal samples are anuran amphibians). The tube is chilled on ice for more than 30 min. The suspension is briefly centrifuged at 1000 × g for 1 s to remove large fragments and the supernatant is transferred into a new tube. In order to concentrate the trophozoites, the tube is centrifuged at 750 x g for 5 min and the supernatant is discarded. The sediment is resuspended with adapted TYI-S-33 medium and kept at 37 °C for 30 min. Then the adapted TYI-S-33 medium is replaced by the new adapted TYI-S-33 medium and kept at 37 °C for 30 min. Then the adapted TYI-S-33 medium is replaced by the new adapted TYI-S-33 medium. And the tube is immediately chilled in ice for 30 min. Then the tube is centrifuged at 2000 × g for 5 min, the supernatant is discarded and the trophozoites are collected from the pellet. The trophozoites are re-suspended with phosphate buffer saline (PBS) (0.65% sodium chloride solution for anuran amphibians) (Fig. 1). This physiological active *Giardia* trophozoite samples could be used immediately for any other research. Trophozoite samples could also be stored at 4 °C for 3 d for further experiment (Fig. 2).

*Giardia* cysts are collected from feces of hosts. Feces could be directly collected from the habitats of the host animals, or be collected by feeding the host animals for several days. Feces samples are re-suspending in water, and filtering the suspension with a 60 mesh and a 200 mesh sieve. Transferred the filtrate into a new centrifuge tube and centrifuged at 1500 × g for 10 min. Then discard supernatant. A 33% zinc sulfate solution with the same volume of sediment is added to re-suspend the sediment, and centrifuged at 1200 × g for 15 min. The supernatant is transferred into a new centrifuge tube and diluted with 4 times the volumes of water and centrifuged again at 1500 × g for 10 min. Then the last four steps are repeated twice to purify the cysts. The precipitated cysts are re-suspended with PBS (Fig. 1). This physiological active *Giardia* cyst samples could be used immediately for any other research. *Giardia* cysts samples could be stored for one month at 4 °C for further experiment.

### Morphological identification

**Fig. 3:**
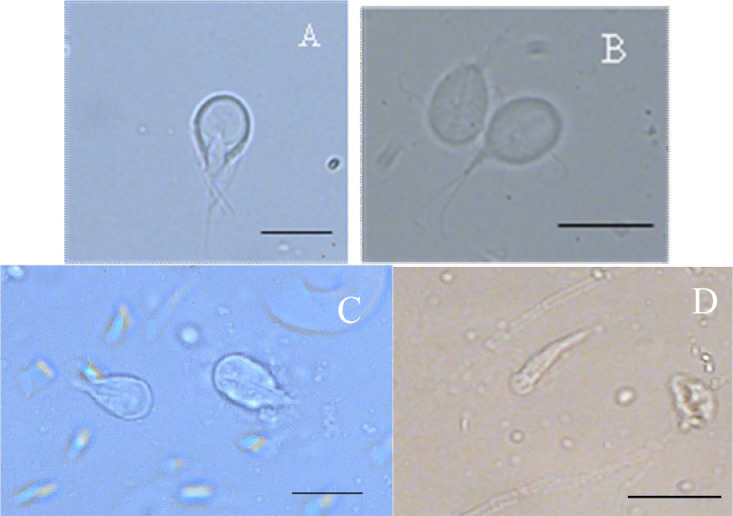
Trophozoites of Giardia species. A Trophozoite of *G. cricetidarum*; B Trophozoite of *G. muris*; C Trophozoite of *G. microti*; D Trophozoite of *G. agilis*[2]. Scale-bars: A, B, C 10 µm; D 20 µm.

**Fig. 4:**
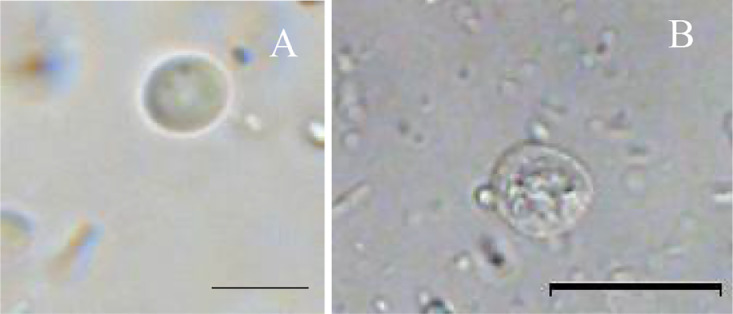
Cysts of Giardia species. A Cyst of *G. muris*; B Cyst of *G. cricetidarum*[1]. Scale-bars: A 10 µm; B 20 µm.

The trophozoite and cyst samples described above are dropped on slides and cover by 20 mm square coverslips. All slides and the whole coverslip should be completely checked. All slides are examined under 40 × and 100 × (with oil immersion) optical microscope. The images are captured by a digital camera. *Giardia* species have a specific pyriform-shaped trophozoites with four pairs of flagella and a ventral disc (Fig. 3), and cysts of *Giardia* are ovoidal (Fig. 4). Trophozoites of different *Giardia* species could be distinguished by their length and width (Table 1).

**Table 1:** The trophozoite sizes of *Giardia* species [1].

Species	Length(mm)	Width(mm)
*G. cricetidarum*	12-18	8-12
*G. intestinalis*	12-15	6-8
*G. muris*	9-12	5-7
*G. microti*	12-15	6-8
*G. agilis*	20-30	4-5
*G. ardeae*	~10	~6.5
*G. psittaci*	~14	~6

### Molecular identification

We designed many primers according to the conserved fragments of *glutamate dehydrogenase, beta-giardin, elongation factor-1 alpha, triose phosphate isomerase* and *SSU rRNA* in all sequenced *Giardia* species. We used all primers to amplify the isolated trophozoites. And, we finally found two specific primers which are applicable to all *Giardia* species. Primer pairs of *Small Subunit rRNA* (*SSU rRNA*): AGCAGCCGCGGTAATTCC and CCTTGTTACGACTTCTCCTTCC (product lengths is approximately 950 bp); *beta-giardin*: GCGAGGAGGTCAAGAAGTC and GAGCGTGTTGACGATCTTGT (product lengths is approximately 500 bp). The trophozoite and cyst samples are kept at 100 °C before PCR. The PCR reactions are set up in PCR Mix (TaKaRa, Tokyo, Japan), 1 µM of each primer and 10 µl treated trophozoites or cysts sample (it is also feasible to use total fecal DNA). Thermocycling conditions are as follows: 94 °C for 10 min followed by 30 cycles of 94 °C for 30 s, 57 °C for 30 s and 72 °C for 90 s, followed by 72 °C for 10 min. The PCR products could be sequenced directly or sequenced after purification. The PCR products are purified using the Gel and PCR clean-up system kit (TaKaRa), and cloned into pMD-19T vectors using TaKaRa pMD-19T VectorCloning Kit (TaKaRa, Tokyo, Japan). The ligation products are transformed into DH5α chemically competent *E. coli*. Colony PCR is carried out with the vector-specific primers provided in the kit, and colonies are selected and Sanger-sequenced using vector-specific forward and reverse primers. The products sequences are analyzed using phylogenetic analysis by maximum likelihood method. Regularly used gene sequences are listed: *SSU rRNA* of *G. agilis* (GenBank: MN227552), *G. cricetidarum* (GenBank: MF185957), *G. muris* (GenBank: MF185956), *G. microti* (GenBank: MF185958), *G. psittaci* (GenBank: AF473853.1), *G. muris* (GenBank: X65063 S53320), *G. ardeae* (GenBank: Z17210 S53313), *G. microti* (GenBank: AF006677), *G. intestinalis* Assemblage A isolate WB (GenBank: M54878 M19), *G. intestinalis* Assemblage B (HG425134), *G. intestinalis* Assemblage E (HG425150); *β-giardin* sequences of *G. cricetidarum* (GenBank: MF185953), *G. agilis* (GenBank: MF185954), *G. microti* (GenBank:MF185955), *G. intestinalis* Assemblage_A (GenBank: KJ363393), *G. intestinalis* Assemblage_B (GenBank: KJ363389), *G. intestinalis* Assemblage_D (GenBank: KJ027418), *G. intestinalis* Assemblage_E (GenBank: KJ363399), *G. intestinalis* Assemblage_F (GenBank: KJ027424), *G. muris* (GenBank: EF455599), *G. muris* (GenBank: AY258618), *G. psittaci* (GenBank: AB714977).

## Declaration of Competing Interest

The authors declare that they have no known competing financial interests or personal relationships that could have appeared to influence the work reported in this paper.
